# Yeast‐Based Biotechnology for Civilian Security

**DOI:** 10.1111/1758-2229.70280

**Published:** 2026-01-29

**Authors:** Justyna Ruchała, Roksolana Vasylyshyn, Maciej Wnuk

**Affiliations:** ^1^ Faculty of Biotechnology, Medical College, University of Rzeszów Rzeszów Poland; ^2^ Department of Molecular Genetics and Biotechnology Institute of Cell Biology, NAS of Ukraine Lviv Ukraine

**Keywords:** biodefense, biotechnology, civilian security, yeasts

## Abstract

Yeasts are remarkably versatile microorganisms whose applications reach far beyond their traditional roles in fermentation. In recent years, they have also emerged as valuable tools in areas related to biosecurity and civilian protection. This paper explores how both conventional and non‐conventional yeast can contribute to the detection, neutralisation, and prevention of biological and chemical threats. We review the use of recombinant yeast cells in biosensors for heavy metals, organic pollutants and endocrine‐disrupting compounds, as well as their role in bioremediation and toxin removal. Special attention is given to the development of yeast‐based vaccine platforms, including RNA and antigen‐display systems using *Saccharomyces cerevisiae* and *Komagataella phaffii*. These technologies illustrate how yeast can bridge biotechnology and security, offering low‐cost, scalable and sustainable solutions. However, practical deployment still faces challenges such as biosensor stability, regulatory barriers for genetically modified strains and the need for standardised calibration. Altogether, yeast biotechnology is positioned as a promising and resilient component of future biodefense and environmental protection strategies, strengthening preparedness in the face of hybrid biological and chemical threats.

## Graphical abstract

Yeasts constitute robust and versatile eukaryotic platforms for biotechnological applications relevant to civilian security. Both native and genetically engineered yeast systems enable sensitive detection of chemical and biological hazards, effective removal of metals and organic pollutants, and detoxification of mycotoxins. In parallel, yeast‐based platforms support vaccine development and anti‐toxin strategies, highlighting their potential as scalable, cost‐effective and sustainable tools for detection, neutralisation and prevention of hybrid threats.

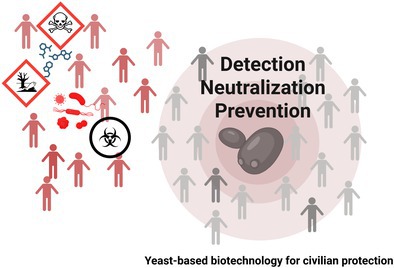

## 1 Introduction

Yeasts belong to a varied group of eukaryotic microorganisms that are part of the fungal kingdom. Primarily unicellular, certain yeast species, dimorphic species such as *Candida albicans* can also develop into multicellular forms known as pseudohyphae or true hyphae, which are crucial in different ecological niches and industries (Liu 2009; Stewart 2014). There are approximately 2200 species belonging to over 150 genera of ascomycetous and basidiomycetous yeasts, but it is estimated that this represents only a small fraction of the species inhabiting the biosphere (Boekhout et al. 2022; Menu et al. 2023). Yeast, long valued in food and beverage production, has gained importance in recent decades as a microbiological cell factory critical to biotechnology (Bourdichon et al. 2012). This includes not only the historic strain *Saccharomyces cerevisiae* , known as brewer's and baker's yeast, but also a wide range of so‐called ‘non‐conventional yeasts’ from the *Saccharomycotina* subphylum, which are expanding the spectrum of industrial applications (Garay et al. 2016; Johnson 2013; Riley et al. 2016; Spencer and Ragout De Spencer 2002). The importance of yeast lies in its naturally diverse metabolic pathways that allow it to perform complex biochemical processes, including fermentation, lipid metabolism and sugar alcohol production, with research showing that biotechnologically important traits like methylotrophy, lipogenesis, xylose fermentation, and the use of cellulose‐ and hemicellulose‐derived sugars are frequently restricted to single phylogenetic clades (Groenewald et al. 2014; Heistinger et al. 2022; Solieri 2021; Wegat et al. 2022). The work by Ruchala et al. (2022) on engineered *Candida famata* producing riboflavin from cheese whey demonstrates the potential for valorization of waste streams, yet also underscores challenges of strain optimization and cost‐effectiveness in non‐lab settings (Ruchala et al. 2022). Yeast's genetic and metabolic diversity makes it irreplaceable in producing a wide range of compounds, serving today as production platforms in key sectors such as traditional food and beverage fermentation, synthesis of bioproducts from biomass, and the production of biofuels and biopharmaceutical molecules (Bourdichon et al. 2012; Fernandes et al. 2023; Haniffadli et al. 2024; Martin and Chan 2024). Native and recombinant yeast producers of lactic acid, as reviewed by Tsaruk et al. (2025), display greater acid tolerance and broader substrate utilisation, highlighting the versatility of non‐conventional yeasts for industrial applications (Tsaruk et al. 2025). At the same time, it should be noted that advances in synthetic biology, including precision genome editing technologies, such as CRISPR‐Cas9, are constantly expanding the range of yeast species useful in biotechnology, enabling metabolic engineering and optimization of their performance (Patra et al. 2021; Xia et al. 2023).

**FIGURE 1:**
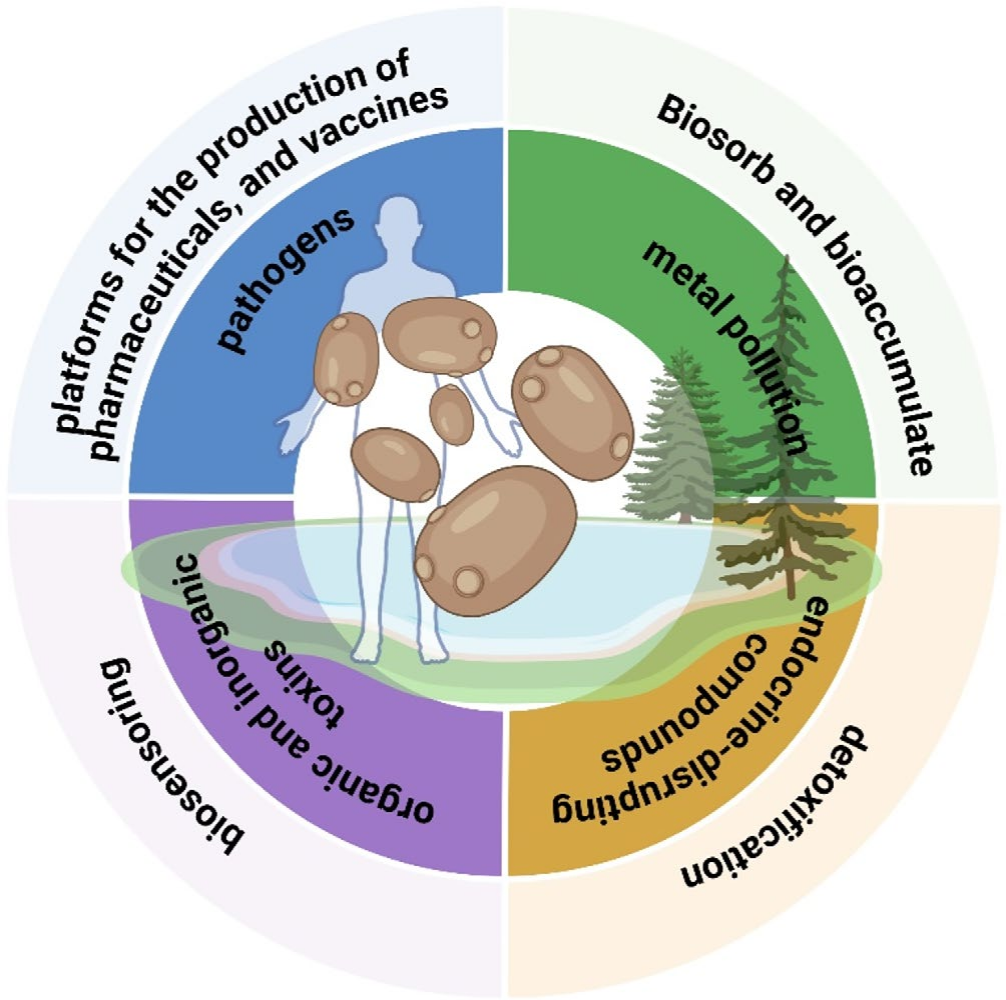
Summary diagram of the use of yeast in protecting the environment and the humans (details are provided below in text).

The unique metabolic characteristics of species and yeast strains, the ease of genetic modification and the low cost of cultivation mean that yeast can also be seriously considered as a ‘platform’ for applications relevant to biosecurity and biodefense. At the same time, the use of yeast‐based biotechnology can be an effective tool in civil defence against hybrid attacks. Hybrid attacks are multifaceted incidents that may combine biological, chemical and technological elements. Their purpose is typically to disrupt critical infrastructure or public health systems rather than to act as purely biological threats (Genini 2025). In the presence of this challenge, yeast‐based systems may contribute to resilience through rapid biosensing and bioremediation technologies. As a biotechnological tool, it contributes to the defence against the biological and chemical elements of these attacks, strengthening the resilience of key state systems (Figure 1).

In this context, biosecurity refers not to containment of pathogens but to the application of biotechnological tools that support the detection and mitigation of biological or chemical threats. The potential of yeast to counter these threats can be exploited at three complementary levels: detection, removal and prevention of future attacks (Aeini et al. 2025; Jarque, Bittner, Blaha, and Hilscherova 2016; Kumar and Kumar 2019). One of the key advantages of using yeast in combating hybrid attacks is its role as a fast and scalable enabling technology capable of securing critical services that are the main target of such destabilising activities. These applications include both critical infrastructure protection and immediate medical response, thereby strengthening a country's resilience. Yeast‐based biosensors help identify attack signatures, distinguishing between natural phenomena and deliberate sabotage by responding to unusual concentrations of substances. They also monitor chemical vectors by detecting trace amounts of unusual chemicals or metabolites in the environment, allowing the direction and type of threat to be identified (Jarque, Bittner, Blaha, and Hilscherova 2016; Smutok et al. 2021). Despite their potential, yeast‐based biosensors still face significant technological limitations. Their performance depends on the viability of living cells, which can be strongly affected by temperature, pH and toxic environmental conditions, limiting stability in field applications (Jarque, Bittner, Blaha, and Hilscherova 2016; Wahid et al. 2023). Signal calibration and reproducibility also remain challenging, as metabolic fluctuations may cause inconsistent fluorescence or luminescence outputs (Jarque, Bittner, Blaha, and Hilscherova 2016). Although freeze‐dried and encapsulated yeast systems have improved shelf life (Chamas et al. 2025), long‐term stability, regulatory constraints on genetically modified strains and sensitivity to environmental stress continue to hinder large‐scale deployment (Chamas et al. 2025; Wahid et al. 2023).

The use of yeast‐based biotechnological tools in preventing hybrid attacks may therefore involve the use of rapid, modular and inexpensive biotechnology to neutralise contamination, reduce response times and provide treatment, ultimately strengthening the resilience of society and the state to destabilisation. In this forward‐looking paper, we discuss various aspects of the potential use of yeast in a range of applications related to the detection, limitation, protection and countering of threats associated with hybrid attacks (Figure 1).

## 2 Yeast in the Protection and Prevention of Environmental Contamination With Metals

Recombinant yeast cells have emerged as powerful tools for biosensing applications due to their ability to be genetically engineered for high sensitivity and specificity in detecting various analytes including hazardous substances (Chamas et al. 2025, 2017; Jarque, Bittner, Blaha, and Hilscherova 2016; Jarque, Bittner, and Hilscherová 2016; Wahid et al. 2023) (Table 1). Yeast is now widely used as an advanced biosensor for monitoring overall environmental toxicity, including drinking water and wastewater (Jarque, Bittner, Blaha, and Hilscherova 2016). Their use is particularly valuable in the rapid and accurate detection of heavy metals (Fan et al. 2022), which is crucial both for public health protection and in the context of countering hybrid attacks on water infrastructure. The mechanism of action of these biosensors is based on genetic engineering, for example, inserting a reporter gene (e.g., responsible for bioluminescence or fluorescence) into the yeast genome. This gene is controlled by promoters activated by specific factors—in this case, by oxidative stress caused by toxic substances or by the activation of cell detoxification pathways. For example, when a water sample comes into contact with toxic concentrations of heavy metals (such as cadmium, arsenic, lead), the promoter is activated and the yeast cell emits a strong light signal (Martin‐Yken 2020). This immediate and visible phenomenon allows for continuous monitoring of water quality at intake points, enabling rapid detection of illegal industrial waste discharge or contamination resulting from a deliberate attack on water supply systems. As a result, the response time to a threat is reduced from days to hours (Chamas et al. 2025; Martin‐Yken 2020; Park et al. 2007; Shepherd et al. 2022). Yeast has also been used in the development of biosensors for the rapid and accurate detection of harmful substances, especially metal ions such as arsenic (Cu2+), copper (As3+), iron (Fe2+), lead (Pb2+) and cadmium (Cd2+) (Fan et al. 2022; Radhika et al. 2005). Yeast‐based biosensors offer instant readings and high accuracy, making them suitable for field use and portable monitoring devices. Recombinant yeast containing specific biomarkers can detect toxic chemicals, including heavy metals and the presence of contaminants is signalled, for example, by the intensity of green fluorescence. Many yeast species exhibit a natural ability to biosorb and bioaccumulate heavy metals, which involves binding them to the cell surface or accumulating them inside the cell. For example, the species *Rhodotorula mucilaginosa* and others of the genus *Rhodotorula* have a significant capacity to accumulate metals, including copper, zinc, lead, chromium and cobalt (Radić et al. 2017). Their biosorption potential can be estimated and exploited to remove these contaminants from solutions. Similarly, *Candida tropicalis* is distinguished by its high bioaccumulation capacity, achieving, for example, up to 94.37% copper bioaccumulation (Radić et al. 2017). Even the well‐known *S. cerevisiae* (brewer's yeast) is effective in the biosorption of copper, lead and zinc, where free amino acids present in the yeast biomass play a key role in the coordination of metal ions. The unique metabolic properties of yeast make it a promising candidate for the bioremediation of metal‐contaminated environments (Fan et al. 2022). *Yarrowia lipolytica* is used in bioremediation due to its ability to produce metal‐binding proteins and pigments, such as melanin, which aid in metal adsorption and nanoparticle formation (Kolhe et al. 2022). Another species, *Geotrichum* sp. CS‐67, has shown a significant ability to accumulate heavy metals, including zinc (Zn^2+^), nickel (Ni^2+^) and copper (Cu^2+^) (He et al. 2022). Furthermore, yeasts isolated from contaminated environments, such as *R. mucilaginosa* from mine waters, are being studied for their ability to remove manganese (Mn2+) ions, confirming their potential in the biotechnological treatment of contaminated water (Ruas et al. 2020). Even *S. cerevisiae* is proposed for the bioremediation of heavy metal contamination in regions affected by intensive mining activity (Zaharia 2017). A suspension of *S. cerevisiae* yeast was studied for the decontamination of radioactive wastewater, demonstrating cell‐wall biosorption and complexation of thorium and uranyl ion (Humelnicu et al. 2004). Yeast offers a versatile set of tools—primarily based on passive biosorption and bioaccumulation mechanisms, complemented by biosensor and bioremediation strategies—making it a valuable component of systems designed to mitigate and monitor metal contamination in the environment. Advances by Sibirny and co‐workers highlight that non‐conventional yeasts can be metabolically engineered to efficiently produce fuels and high‐value chemicals, with traits that support operation under demanding process conditions (Sibirny 2023). Electrochemical and microelectrode biosensors are increasingly complemented by AI‐assisted signal analysis; these advances are applicable to yeast‐based formats and can enhance precision and portability (Chamas et al. 2025; Liu et al. 2025; Zhou et al. 2024).

**TABLE 1:** Yeast‐based biosensors and detection platforms for biological and chemical threats.

Organism/system	Detected target	Principle/mechanism	Analytical features or application	References
Environmental and chemical biosensing and bioremediation				
Recombinant *S. cerevisiae* (oxidative‐stress and metal‐specific promoter reporters)	Cd^2+^, As^3+^, Pb^2+^, Cu^2+^, Fe^2+^	Reporter genes (luciferase/GFP/*lacZ*) controlled by general detoxification (e.g., *GRE2*, *YCF1*) or metal‐responsive (*CUP1*, *ACR3*) promoters, with reporter‐gene activation independent of oxidative stress sensors	Fluorescence or luminescence upon exposure to toxic metals; broad‐ and metal‐specific response for rapid monitoring of water quality	Chamas et al. (2025), Jarque, Bittner, Blaha, and Hilscherova (2016), Martin‐Yken (2020), Park et al. (2007), Shepherd et al. (2022)
*S. cerevisiae* (biosensor constructs)	Heavy metals (Cd, Pb, Cu, Fe, As)	Promoter activation by ROS → light emission	Continuous water quality control in critical infrastructure	Fan et al. (2022), Radhika et al. (2005)
*R. mucilaginosa* , *C. tropicalis* , *Y. lipolytica*, *Geotrichum* sp.	Heavy metals (Cu, Zn, Pb, Cr, Co, Ni)	Biosorption and bioaccumulation; cell wall binding and pigment‐mediated adsorption	Quantitative removal of metal ions; combined sensing‐remediation function	He et al. (2022), Kolhe et al. (2022), Radić et al. (2017), Ruas et al. (2020)
*S. cerevisiae* electrochemical biosensors (AI‐assisted, screen‐printed electrode type)	Cu^2+^, Pb^2+^, Cd^2+^, toxic metal ions	Whole‐cell electrochemical transduction on disposable screen‐printed electrodes; bio‐electrochemical recognition with AI‐assisted signal processing	Low‐cost, portable, single‐use biosensors enabling real‐time field deployment and high‐precision detection	Chamas et al. (2025), Liu et al. (2025), Zhou et al. (2024)
*S. cerevisiae* (ERG‐promoter NanoLuc biosensor)	Azole fungicide tebuconazole (TEB)	Reporter gene (NanoLuc) under ergosterol‐biosynthesis promoters (*ERG3, ERG6, ERG11, ERG25*) activated upon sterol‐pathway inhibition	Specific detection of tebuconazole ≥ 5 μg L^−1^; high selectivity against other azoles; environmental monitoring of agrochemical contamination	Mendes et al. (2024)
*S. cerevisiae* (YES and YAS assays; miniaturised HTS formats)	Estrogenic and androgenic endocrine‐disrupting compounds (EDCs)	*lacZ*, luciferase, or GFP reporters under oestrogen‐responsive promoters or human receptor elements (ERα, AR)	Rapid, automated detection of EDCs; compatible with fluorometric and robotic high‐throughput screening	Gregório et al. (2024), Rajasärkkä and Virta (2013)
Recombinant *S. cerevisiae* expressing human receptors	ER, PR, AR ligands	Fluorescent or luciferase reporters controlled by human nuclear receptor response elements	Compatible with liquid cultures and HPTLC coupling for multi‐receptor screening	Abu‐Rmailah et al. (2024)
Ready‐to‐use Yeast Assays (RYAs; frozen or lyophilised *S. cerevisiae* biosensors)	Estrogenic and androgenic endocrine‐disrupting compounds	Pre‐stabilised yeast biosensors in lyophilised form	Portable field kits; minimal equipment required	Jarque, Bittner, and Hilscherová (2016)
Self‐bioluminescent multiplex *S. cerevisiae* strains	Bisphenol A, BPA analogues	Multi‐receptor (ERα, ERβ, PR, AR) autobioluminescent system	Multiplex analysis of endocrine activity	Huang et al. (2024)
Recombinant *S. cerevisiae* + HPLC (HPLC–Bioassay)	Oestrogen‐like pollutants in environmental samples	Coupling of chromatographic fractionation with recombinant yeast screening	Precise identification of estrogenic compounds in complex mixtures	Iwasaki et al. (2004)
*S. cerevisiae* bioassay combined with solid‐phase extraction (SPE)	Endocrine disruptors in water	Concentration step prior to yeast‐based detection	Increased sensitivity; detection of PR inhibition	Li et al. (2006)
Immobilised *S. cerevisiae* cells (gelatin matrix)	Estrogenic/androgenic activity	Immobilisation maintains viability and signal responsiveness	Continuous environmental monitoring; improved long‐term stability	Li et al. (2006)
*S. cerevisiae* (cell surface display biosensor systems)	Various environmental contaminants and toxic compounds	Functional proteins or enzymes fused to anchoring motifs (GPI‐, Flo1p‐ or Pir‐based) and displayed on the yeast cell wall; enables direct binding, catalysis or detection at the cell surface	Reusable whole‐cell biosensor or biocatalyst; improved stability under harsh conditions; applicable to pollutant removal and bioconversion	Zhang et al. (2022)
Yeast‐based bioremediation and catalytic degradation systems				
*S. cerevisiae* displaying metallothionein (SMT) from *Solanum nigrum*	Cd^2+^	Surface display via α‐agglutinin of metal‐binding peptides	Remediation by high‐affinity Cd^2+^ adsorption; reusable biosorbent	Wei et al. (2016)
*S. cerevisiae* displaying MerR regulatory protein	Hg^2+^	Surface display of Hg^2+^‐binding regulator MerR	Enhanced Hg^2+^ tolerance and adsorption; remediation focus	Wei et al. (2018)
*S. cerevisiae* displaying cutinase FsC (*Fusarium solani pisi*)	Parabens	Surface enzyme catalysis via display of FsC	Enzymatic degradation of parabens in water; remediation	Zhu and Wei (2019)
*S. cerevisiae* displaying urethanase UreA (*Micrococcus* sp.)	Ethyl carbamate	Surface enzyme catalysis via display of UreA	Biodegradation of EC in fermented beverages; food safety remediation	Han et al. (2022)
Magnetically modified *S. cerevisiae*	Heavy metals, radionuclides	Magnetised cell surface allows for magnetic separation and quantification	Rapid detection and recovery of bound contaminants	Safarik et al. (2014)
Recombinant *S. cerevisiae* or *Y. lipolytica*	Organophosphates, mycotoxins	Expression of heterologous detoxification enzymes; signal via metabolic activity	Whole‐cell catalysts for detection and degradation of organic pollutants	Chlebicz and Śliżewska (2020), Zou et al. (2015)
Biomedical and immunological yeast‐based detection platforms				
*S. cerevisiae* and *K. phaffii* (Whole Yeast Vaccine, WYV, platforms)	Antigens (proteins, RNA constructs; viral antigens HBV, HPV)	Genetically modified yeast cells expressing recombinant antigens; scalable bioreactor production	Dual detection–immunisation concept bridging biotechnology, biodefense and industrial vaccine manufacturing	Bazan et al. (2014), Kumar and Kumar (2019),Wen et al. (2014), Silva et al. (2021), Tan et al. (2022)
*S. cerevisiae* extracellular vesicles (YEVs)	RNA, miRNA, immunogenic cargo	Natural or engineered EVs delivering nucleic acids	Immunotherapeutic or adjuvant roles; tumour inhibition; biocompatibility	Higuchi et al. (2023), Morishita et al. (2023), Yuan et al. (2024)
*C. albicans* extracellular vesicles (YEVs vs. HEVs)	EV subclasses (protective vs. cytotoxic)	Comparative EV profiling	Importance of EV characterisation before clinical use	Martínez‐López et al. (2024)
*S. cerevisiae* field‐deployable biosensors (test‐strip format)	Broad range of contaminants, hybrid threats	Luminescence or colour change as visual output	Simple, disposable, rapid‐response biosensors for on‐site monitoring	Chamas et al. (2017), Jarque, Bittner, and Hilscherová (2016), Wahid et al. (2023)

*Note:* Overview of yeast‐based biosensing systems designed for detection and monitoring of chemical pollutants and biological contaminants.

## 3 Yeast‐Based Tools in the Fight Against Threats Caused by Harmful Organic Compounds

Yeast‐based tools can play a special role in detecting the presence of toxic organic compounds and contributing to their mitigation, primarily through biosorption, biotransformation, or enzymatic degradation rather than direct chemical neutralisation (Bobate et al. 2023). Yeasts can mitigate toxic contamination through multiple mechanisms, including passive biosorption to cell wall polysaccharides, intracellular bioaccumulation and enzyme‐driven biotransformation (Table 2). Recent studies report the degradation of xenobiotics such as organophosphates and mycotoxins by engineered *S. cerevisiae* and *Y. lipolytica*, demonstrating their growing potential in detoxification and environmental remediation (Chlebicz and Śliżewska 2020; Zou et al. 2015). The most commonly described example of the use of yeast‐based bioassays is the assessment of the potential endocrine activity of transformation products formed (Bobate et al. 2023), for example, during the ozonation of water containing pesticides (Westlund et al. 2018). Such tests help to assess the environmental risks associated with intentional or accidental contamination of drinking water. Another innovative approach is the use of magnetically modified yeast cells as active elements in biosensors. Their magnetic modification allows for easy separation and detection of not only organic xenobiotics but also heavy metal ions and radionuclides (Safarik et al. 2014). As with metals, yeast is a valuable component of bioremediation strategies for xenobiotics and pesticides (Bobate et al. 2023). Genetically modified yeast can serve as whole‐cell catalysts or hosts for heterologous expression of biodegradation enzymes, effectively removing toxic pesticide residues from soil and water. For example, *S. cerevisiae* has potential for the biodegradation and bioaccumulation of nitrophenols, contributing to the reduction of the toxicity of these compounds (Pintilie et al. 2016). The ability of yeast to biosorb and detoxify stems from its high adsorption capacity, which includes mechanisms such as physical adsorption, precipitation, complexation, ion exchange and redox reactions (Aeini et al. 2025). Currently, the practical application of this technological solution can be observed in the food industry during alcoholic fermentation, where yeast can reduce the concentration of pesticide residues, increasing the safety of wine production (Becerra et al. 2023). In addition, yeast is used in wastewater treatment, demonstrating great versatility and the ability to process various sources of organic carbon and remove contaminants even under non‐sterile conditions (Mohiuddin et al. 2024). Despite promising solutions, fully exploiting the potential of yeast requires overcoming certain challenges. Issues related to scaling up in situ degradation processes, safety concerns during field trials and limited attention to certain types of pesticides need to be addressed. Furthermore, the effectiveness of yeast in wastewater treatment and contaminant removal is variable and depends on the specific properties of the wastewater and the type of contaminants present. In summary, yeast offers a sustainable and environmentally friendly approach to the detection and removal of xenobiotics and pesticides, with applications ranging from advanced biosensors to effective bioremediation in various environmental contexts.

**TABLE 2:** Yeast‐based systems for detoxification and bioremediation of xenobiotics.

Organism/system	Target compound(s)	Detoxification/mechanism	Efficiency/key findings	References
Heavy metals and radionuclides				
*R. mucilaginosa*	Cu^2+^, Zn^2+^, Pb^2+^, Cr^6+^, Co^2+^	Biosorption and intracellular bioaccumulation	High uptake under optimised lab conditions; efficiency depends on pH, competing ions and biomass dose	Radić et al. (2017), Ruas et al. (2020)
*C. tropicalis*	Cu^2+^	Surface binding and intracellular accumulation	High Cu^2+^ removal in batch assays; applicable for wastewater decontamination	Radić et al. (2017)
*S. cerevisiae*	Cu^2+^, Pb^2+^, Zn^2+^, Th^4+^, UO_2_^2+^	Cell‐wall biosorption via phosphoryl, carboxyl, amine and hydroxyl groups; uranyl complexation	Broad binding spectrum for metals and radionuclides; relevant to mining effluents	Humelnicu et al. (2004), Zaharia (2017)
*Y. lipolytica*	Ni^2+^, Cu^2+^, Zn^2+^	Melanin‐linked and protein‐mediated metal binding	Strong biosorption by wastewater isolates; matrix‐dependent performance	Kolhe et al. (2022)
*Geotrichum* sp. CS‐67	Zn^2+^, Ni^2+^, Cu^2+^	Active bioaccumulation via membrane transport systems	High uptake in lab reactors; potential for industrial effluent treatment	He et al. (2022)
Organic pollutants and pesticides				
*S. cerevisiae, Y. lipolytica* (engineered)	Organophosphates, nitrophenols, pesticides	Enzyme‐mediated biotransformation (organophosphatases, oxidoreductases, laccases)	Efficient degradation of organophosphorus and phenolic residues in soil and water	Pintilie et al. (2016), Zou et al. (2015)
*S. cerevisiae* (surface display of organophosphorus hydrolase, OPH)	Organophosphates (e.g., paraoxon)	Yeast surface‐anchored OPH catalysis	Functional OPH displayed on *S. cerevisiae* MT8‐1; hydrolysis of OPs demonstrated on whole cells	Takayama et al. (2006)
*Komagataella phaffii* secreting Trametes laccase	Synthetic dyes, phenolics	Secreted laccase oxidative degradation	High decolorization efficiencies for multiple azo/anthraquinone dyes in wastewater models	Jia et al. (2022)
*S. cerevisiae* (bioassay with detox function)	Organic xenobiotics, endocrine disruptors	Biosorption with contribution of enzymatic transformation	Reduced toxicity in vitro; mechanism primarily adsorption	Bobate et al. (2023), Chlebicz and Śliżewska (2020)
*S. cerevisiae* (wine fermentation strains)	Pesticide residues	Passive adsorption during fermentation	Lower pesticide residues in final product; effect compound‐ and matrix‐dependent	Becerra et al. (2023)
Magnetically modified *S. cerevisiae*	Metals, organic xenobiotics, radionuclides	Magnetically assisted biosorption and recovery	Easy separation and reuse; hybrid detection–removal functionality	Safarik et al. (2014)
*Y. lipolytica*, *Candida* spp. (wastewater isolates)	Diverse organic pollutants	Biosorption, precipitation, redox interactions	Effective removal under non‐sterile wastewater conditions; pollutant‐specific efficiency	Aeini et al. (2025), Mohiuddin et al. (2024)
Mycotoxins and food/feed detoxification				
*S. cerevisiae* + *Lactobacillus* sp. consortium	Deoxynivalenol (DON)	Combined adsorption and enzymatic conversion	Synergistic detoxification and improved animal health indicators	Azizi et al. (2021)
*S. cerevisiae* cell‐wall preparations/adsorbents	DON, AFB1, T‐2, ZEA, fumonisins	Binding by mannoproteins and β‐glucans (MOS/β‐glucans)	Reduced bioavailability in animal trials; binding can be partly reversible under GI‐like conditions	Marković et al. (2023), Pang et al. (2022), Xu et al. (2023)
*S. cerevisiae* (biotransformation strains)	AFB1, ZEA, T‐2 toxin	Enzymatic conversion to less‐toxic or more excretable metabolites	Demonstrated in vitro and in vivo; species‐ and enzyme‐dependent efficacy	Pfliegler et al. (2015), Schatzmayr et al. (2006)
*S. cerevisiae RC016, RC008*	Aflatoxin B_1_	Non‐covalent adsorption via mannoproteins and β‐glucans	Up to ~80% AFB_1_ removal at acidic pH; binding partially retained under simulated gastrointestinal conditions	Armando et al. (2011)
*S. cerevisiae* cell walls/inactive biomass	Ochratoxin A (OTA)	Adsorption by β‐glucans/mannoproteins of the cell wall	Rapid OTA removal from wine/grape must; mechanism confirmed for yeast hulls/cell wall fractions	Caridi et al. (2012)
*S. cerevisiae* (wine/juice isolates)	Patulin	Predominantly adsorption to cell wall; partial biodegradation	Significant PAT decrease in apple juice; strain‐ and matrix‐dependence quantified	Guo et al. (2012), Jiang et al. (2024)

*Note:* Overview of yeast‐driven strategies emphasising active removal, adsorption or enzymatic transformation of toxic xenobiotics in environmental and industrial contexts.

### 3.1 Yeast Platform for Mycotoxins in Food/Feed Detoxification

Yeasts, particularly *S. cerevisiae* strains, play a key role in improving the safety and quality of animal feed by binding and reducing the bioavailability of various toxins, mainly mycotoxins, through cell‐wall adsorption mechanisms (Table 2). Their ability to bind and detoxify these harmful substances, combined with the benefits of being a feed additive, makes them a valuable tool in veterinary medicine and agriculture. Studies have shown that yeast is effective in removing a wide spectrum of mycotoxins. For example, the strain *S. cerevisiae* SC1221 shows a strong ability to remove T‐2 toxin from feed (Zou et al. 2015). The main mechanism in this case is physical binding rather than biotransformation, leading to a significant reduction in toxin after incubation. In the case of aflatoxin B1, yeast strains such as *S. cerevisiae* RC016 and RC008, isolated from the animal environment, have a high capacity to bind this toxin (Armando et al. 2011). Importantly, these strains are able to survive in the gastrointestinal tract, making them promising candidates for inclusion in feed. Yeast is also used to reduce deoxynivalenol (DON), often in synergy—for example, by combining yeast cell walls with *Lactobacillus* strains, which enhances the detoxification process and improves animal health (Azizi et al. 2021). Overall, various strains of *S. cerevisiae* demonstrate the ability to detoxify many mycotoxins, including aflatoxin B1, DON, fumonisins, T‐2 toxin and zearalenone, achieving a significant reduction in their concentration (Chlebicz and Śliżewska 2020; Xu et al. 2023).

The effectiveness of yeast in neutralising toxins results from several key mechanisms of action: physical binding (biosorption), biotransformation and competitive exclusion. However, the most commonly observed mechanism is the binding of mycotoxins by yeast cell walls, rich in mannan oligosaccharides (MOS) and β‐glucans (Zou et al. 2015). This reduces their bioavailability and toxicity by preventing their absorption into the body. Some yeast strains also have a natural ability to biotransform mycotoxins into less toxic metabolites (Pfliegler et al. 2015; Schatzmayr et al. 2006). Competitive exclusion, on the other hand, involves the direct inhibition of the growth of pathogenic bacteria by yeast, reducing the negative effects of exposure to mycotoxins (Oztekin et al. 2023). The use of yeast goes beyond detoxification alone. Yeast and its derivatives are commonly used as feed additives to improve growth, nutrient absorption and overall animal health while reducing the need for antibiotics. Thanks to its properties, yeast acts as both a probiotic and a prebiotic, strengthening intestinal health and limiting the colonisation of pathogens, which is particularly beneficial in environments contaminated with toxins (Marković et al. 2023; Pang et al. 2022). It should be noted that the use of yeast, especially *S. cerevisiae* , is a sustainable and effective strategy for mitigating the adverse effects of mycotoxins, contributing to improved health, performance and safety of livestock.

### 3.2 Yeast Platform for Organic Pollutants and Pesticides

Yeast‐based bioassays are a widely used and highly effective method for detecting and quantifying endocrine‐disrupting compounds (EDCs), that is, substances that disrupt the endocrine system (Table 2). Their popularity stems from their high sensitivity, repeatability and cost‐effectiveness (Gregório et al. 2024). They use yeast cells as test platforms in which the presence of EDCs leads to a measurable signal. The best‐known example is the Yeast Oestrogen Screen (YES) Assay. This test uses *S. cerevisiae* cells that contain the lacZ reporter gene under the control of an oestrogen‐sensitive promoter (Gregório et al. 2024). Under the influence of oestrogenic compounds, these cells produce the enzyme β‐galactosidase, whose activity can be quickly measured using a fluorescent substrate. This method is extremely fast, providing results within 2 h, and is highly sensitive, with an EC50 of 0.17 nM for 17β‐oestradiol, for example. To increase precision, yeast biosensors expressing human hormone receptors and using fluorescent proteins as reporter genes have also been developed, enabling analysis in both liquid cultures and in combination with thin‐layer chromatography (HPTLC) (Abu‐Rmailah et al. 2024). One advance is that the sensitivity and modularity of yeast have allowed for the miniaturisation of tests towards high‐throughput (HTS). Analyses based on nuclear receptors in yeast cells have been reduced to 384‐ and 1536‐well formats, enabling simultaneous and rapid testing of multiple samples using different reporter systems (e.g., β‐galactosidase, luciferase or fluorescent proteins) (Rajasärkkä and Virta 2013). For field research, analyses on frozen and dried yeast (*Ready‐to‐use Yeast Assays*, RYAs) have been developed. These ready‐to‐use kits significantly reduce the need for additional equipment, enabling rapid assessment of oestrogenic and androgenic potential directly in the field (Jarque, Bittner, and Hilscherová 2016). Innovative platforms using autobioluminescent yeast strains allow for multiplex analysis, that is, simultaneous testing of compounds that modify various endocrine receptors (e.g., ERα, ERβ, PR, AR) (Huang et al. 2024). This platform is used to test compounds such as Bisphenol A and its alternatives (Huang et al. 2024). In order to analyse complex environmental samples, yeast bioassays are combined with advanced analytical techniques. The combination of genetically modified yeast with high‐performance liquid chromatography (HPLC‐Bioassay) allows for the precise separation and identification of oestrogen‐like compounds in environmental samples (Iwasaki et al. 2004). In turn, the use of solid‐phase extraction (SPE) prior to yeast analysis increases the sensitivity of detection of endocrine disruptors in complex matrices, as demonstrated by the detection of inhibition of human progesterone receptor activity in water samples (Li et al. 2006). Furthermore, immobilised yeast cells (e.g., in gelatin matrices) retain their viability and sensitivity for a longer period of time, making them ideal for continuous environmental monitoring of oestrogenic and androgenic activity in field applications (Li et al. 2006). Biosensors for warfare‐relevant protein toxins (e.g., ricin, anthrax toxin, botulinum) are well documented in general biosensing literature. Although yeast‐based systems for the direct detection of these toxins have not yet been reported, the modularity and adaptability of yeast biosensors suggest strong potential for such applications (Pohanka 2023). For instance, yeast biosensors have been engineered to detect various chemicals and metabolites, indicating that similar approaches could be tailored for specific protein toxins (Asemoloye and Marchisio 2023; Guenther et al. 2019; Jarque, Bittner, Blaha, and Hilscherova 2016; Tang et al. 2023). In short, yeast‐based tools represent versatile and sustainable biotechnological approaches for addressing chemical and biological threats, ranging from advanced detection to adsorption‐ or enzyme‐based mitigation of hazardous compounds. As demonstrated, yeast biosensors, including systems such as the YES Assay, provide rapid, sensitive and miniaturised methods for detecting endocrine‐disrupting compounds (EDCs) and other toxins, and their modularity allows for integration with advanced chromatography for precise environmental analysis. At the same time, yeast itself, often genetically modified, serves as a key component in bioremediation and detoxification strategies—from removing pesticides from soil and water to neutralising mycotoxins in animal feed, which directly translates into improved food safety and public health.

## 4 Yeasts as Producers of Anti‐Toxin Therapies and Vaccines

Yeasts such as *S. cerevisiae* and *Pichia pastoris* (recently named as *Komagataella phaffii*) have emerged as significant platforms for the production of pharmaceuticals, including anti‐toxin therapies and vaccines (Kumar 2025; Wen et al. 2014) (Table 1). *S. cerevisiae* and *K. phaffii* have proven to be effective systems for the mass and scalable production of recombinant antigen proteins (Kumar 2025; Wen et al. 2014). Their success is confirmed by the fact that they are used to produce licensed and widely used vaccines against hepatitis B and human papillomavirus (HPV) (Kumar 2025; Wen et al. 2014). In addition to the production of antigens themselves, whole yeast cells, in so‐called whole yeast vaccines (WYVs), serve as effective vehicles for delivering antigens (proteins or nucleic acids) directly to antigen‐presenting cells (APCs), promoting a strong and targeted immune response (Bazan et al. 2014; Silva et al. 2021; Tan et al. 2022). Some yeast strains produce natural killer toxins that exhibit strong antimicrobial properties against pathogenic yeasts, fungi and bacteria (Georgescu et al. 2024; Klassen et al. 2017; Magliani et al. 2005). These toxins are inspiring the development of new therapies. Protective antibodies (KT‐Abs—Killer Toxin‐like Antibodies) have been developed that mimic the action of killer toxins and exhibit therapeutic activity against various infections (Magliani et al. 2005). The ability of yeast to synthesise such specific molecules paves the way for the development of innovative and targeted drugs (Table 1). Yeast is emerging as a promising vehicle for RNA‐based vaccine delivery taking advantage of their natural ability to transport nucleic acids and modulate immune responses (Silva et al. 2021; Tsaruk et al. 2025). Silva et al. (2021) review multiple strategies in which whole yeast cells or yeast‐derived molecules are genetically modified to carry mRNA or constructs encoding antigens and stimulate immune activation. Furthermore, oral vaccines using surface‐exposed antigens in yeast have demonstrated immune responses in animal models (Austriaco 2023; Lei et al. 2020). Similarly, a surface‐exposed yeast spore platform has recently been proposed to enhance stability in the gastrointestinal tract (Si et al. 2025). However, translating yeast vaccine concepts into effective medical or veterinary applications still poses significant challenges. Antigen stability, delivery efficiency and immune activation remain limiting factors, particularly for oral formulations. For example, Austriaco (2023) emphasise that low pH, bile salts and digestive enzymes can disrupt antigen integrity and reduce vaccine efficacy in oral yeast delivery systems. Therefore, further optimization of surface presentation methods, encapsulation technologies, and strain selection is necessary to improve immunogenicity and protective efficacy. In addition, genetic engineering enables the creation of yeast expressing viral or cancer antigens. Such yeast, used as therapeutic vaccines, has the potential to stimulate a specific T‐cell response, offering promising treatments for chronic diseases such as cancer and chronic viral infections (Ardiani et al. 2010; Wang et al. 2018). In this way, yeast is cementing its position as a versatile and sustainable foundation for the next generation of drugs and vaccines. In recent years, concrete evidence has emerged that yeast‐derived extracellular vesicles (YEVs) can be effectively used in the context of immunotherapy and vaccines. For example, EVs from *S. cerevisiae* loaded with microRNA or larger mRNA have been used to inhibit tumour growth in mouse models, while maintaining biocompatibility and low immunogenicity (Yuan et al. 2024).

Another example is YEVs from *S. cerevisiae* used as adjuvants—after local administration, they target lymph nodes (B‐cell zones), inducing the expression of proinflammatory cytokines and costimulatory markers on dendritic cells and macrophages, which can support a strong immune response (Morishita et al. 2023).

Furthermore, EVs from baker's yeast stimulate immune cell maturation (e.g., expression of CD40, CD80, CD86) and the production of TNF‐α and IL‐6 (Higuchi et al. 2023). On the other hand, research on *C. albicans* shows that different types of EVs (yeast form YEV vs. fungal form HEV) can produce very different effects—from high protection to harmful reactions, indicating the need for precise characterisation of EV composition and function before clinical applications (Martínez‐López et al. 2024).

## 5 Conclusion and Future Perspective

The comprehensiveness of yeast, crucial in pharmacy, also extends to the area of safety and early warning systems, due to its unique ability to combine a living organism with a digital signal. Their ease of use in the field is a significant advantage; they can be implemented in simple, often disposable devices such as test strips, where a change in luminescence or colour provides an immediate warning signal. Further research should focus on increasing the sensitivity and specificity of these biosensors and developing innovative, miniature platforms for their implementation, which will enable even wider and more effective use of biological systems in field diagnostics and environmental monitoring. In summary, yeast is a key, multifunctional biotechnological platform that opens up new possibilities in the fight against bioterrorism and chemical and biological elements of hybrid attacks (Figure 1). Their unique characteristics, combined with ease of genetic modification and scalability of production, enable the implementation of effective detection, neutralisation and prevention strategies, providing a sustainable and economical element in building the resilience of the state and society to the complex challenges of the present century. Future research should prioritise improving the scalability, reproducibility and long‐term stability of yeast‐based biosystems. Particular emphasis should be placed on standardising calibration procedures for living‐cell biosensors and ensuring regulatory compliance for genetically engineered strains (Chamas et al. 2017; Jarque, Bittner, Blaha, and Hilscherova 2016; Wahid et al. 2023).

The translation of these systems from laboratory to field applications will require safe containment strategies and clear regulatory frameworks to minimise ecological risks. Continued integration of yeast‐based platforms with microfluidic and digital sensing technologies could further enhance their precision and responsiveness in real‐world monitoring and biodefense applications.

## Author Contributions

J.R. conceptualization, co‐wrote and edited the draft of the manuscript. R.V. tables and co‐wrote and edited the draft of the manuscript. M.W. conceptualization, co‐wrote and edited the draft of the manuscript.

## Conflicts of Interest

The authors declare no conflicts of interest.

## Data Availability

Data sharing not applicable to this article as no datasets were generated or analysed during the current study.
